# Bulbar Conjunctival and Tenon's Layer Thickness Measurement using Optical Coherence Tomography

**DOI:** 10.5005/jp-journals-10008-1163

**Published:** 2014-06-12

**Authors:** J Howlett, K Vahdani, J Rossiter

**Affiliations:** Department of Ophthalmology, Musgrove Park Hospital, Taunton, Somerset, United Kingdom; Department of Ophthalmology, Musgrove Park Hospital, Taunton, Somerset, United Kingdom; Department of Ophthalmology, Musgrove Park Hospital, Taunton, Somerset, United Kingdom

**Keywords:** Tenon's layer thickne ss, Bulbar conjunc tival thickness, Optical coherence tomography.

## Abstract

**Purpose:** Observations made during glaucoma filtering surgery (trabeculectomy) suggest variability in the thickness of the bulbar conjunctiva and Tenon's layers between individuals. We propose that this could infuence the final bleb morphology and function.

We designed a pilot study to assess this using optical coherence tomography (OCT) to measure bulbar conjunctival and Tenon's layer thickness.

**Materials and methods:** A total of 67 eyes of 48 individuals were scanned using an optovue Mode RT100 version 2.0 OCT machine. Cross-line CAM-L scans were taken and the com bined bulbar conjunctival and Tenon's layer thickness was measured 3 mm above the superior limbus. Conjunctival and Tenon's layers appeared as a hyper-refective section as opposed to the hypo refective underlying sclera. Measurements were taken using the inbuilt review software.

**Results:** The age ranged from 23 to 91 years. There were 20 mal e s and 28 females. The mean conjunctival and Tenon's layer thick ness was 393 ± 67 microns (mean ± SD) ranging from 194 to 573 microns.

**Conclusion:** Optical coherence tomography conjunctival and Tenon's layer thickness measurements appear to vary significantly between individuals. We postulate that this could infuence the final bleb morphology and may predict the risk of bleb encapsulation and failure or thin avascular blebs. Further assessment could establish cut-offs on which patients should receive intraoperative antimetabolites and/or Tenon's layer excision.

**How to cite this article:** Howlett J, Vahdani K, Rossiter J. Bulbar Conjunctival and Tenon's Layer Thickness Measurement using Optical Coherence Tomography. J Curr Glaucoma Pract 2014;8(2):63-66.

## INTRODUCTION

Trabeculectomy fails to control intraocular pressure (IOP) in 10 to 30% of patients.^1^ Glaucoma filtration surgery's success is limited by postoperative scarring leading to fap fibrosis and bleb failure^2^ with subconjunctival fibrosis being the most common cause of bleb failure.^3^ At the other end of the spectrum there are thin avascular blebs. Hu et al^4^ found that blebs with a large avascular area were associated with a higher risk of bleb leakage.^4^ In patients who did not receive antifibrotics leaks have been reported in 3.3% of patients and the fluorouracil filtering surgery study group reported late onset leaks in 7% of eyes,^5^ although bleb leakage rates are very variable in the literature. Bleb leakage has been found to increase the risk of bleb related infection 25.8 times^6^ which can have devastating effects on vision. This is relevant as perhaps 5 Fluorouracil (5-FU) or mitomycin C (MMC) should not be used in patients with lower conjunctival thick nesses. Our observations made during glaucoma filtering surgery suggest variability in the thickness of the bulbar conjunctiva and Tenon's layers between individuals which we propose could infuence the healing response and therefore the final bleb morphology and function.^7^

Optical coherence tomography (OCT) can provide detailed cross-sectional images of internal structures and has several uses in examining the anterior segment. Singh et al^8^ has used anterior OCT to image trabeculectomy blebs post operatively allowing detail of bleb morphology to be seen which was not visible on the slit-lamp and Gumus et al has used OCT to diag nose and monitor conju nct ivo chalasis.^9^ A nterior seg ment OCT can view the entire anter ior chamber and hence can be used to look at the anterior chamber angle, helping with the diagnosis of narrow angles. Using OCT to examine the conjunctival and Tenon's layer thickness could potentially help predict the success of trabeculectomy surgery which could help tailor glaucoma surgery to the individual.

The bulbar conjunctiva is translucent and composed of a superficial epithelium consisting of two to five layers of stratified columnar cells.^11^ These layers are usually well aligned, and hence, incident light is scattered less creating a hypo refective layer on OCT. The underlying conjunctival stroma consists of irregular fibers, perfused blood vessels, cystic spaces and Inflammatory cells. The stroma highly scatters light and therefore appears hyper-refective. Tenon's layer is mainly irregular fibers and therefore, has high refectivity similar to conjunctival stroma on OCT, which becomes progressively less refective.^12^

Previous studies have looked at thickness of the conjunctiva or conjunctival epithelium using OCT but to our knowledge there has not been a study examining the combined thickness of the conjunctiva and Tenon's layer or of the conjunctiva superiorly where trabeculectomy surgery is routinely performed. Feng et al^13^ used OCT to measure the thickness of temporal bulbar conjunctival epithelium *in vivo* 3 to 5 mm from the limbus in a Canadian population. In 13 subjects aged between 20 and 36 years they found the bulbar conjunctival epithelium to be 44.9 ± 3.4 μm thick. Francoz et al^14^ also measured bulbar conjunctival epithelial thickness using spectral-domain OCT. He found that age had no significant effect on the epithelial thickness but that the mea n conju nct ival epithelial th ick ness was significantly thicker in those with dry eye or on intraocular pressure lowering medication.

Zhang et al^12^ measured bulbar conjunctival thickness using cirrus OCT in 18 normal subjects from a Chinese popu lation with an average age 43.3. They found the lower temporal epithelial conjunctival thickness to be 47.3 ± 8.4 μm, conjunctival stroma 190 ± 47.5 μm and full conjunctival thickness 238.8 ± 51.1 μm. On cirrus OCT they found that 3 mm from limbus they could demarcate conju nctival stroma and Tenons.^12^ Histological analysis by Choetal found that the average thickness of anterior Tenon's capsule on enucleated eyes to be 234 μm, although we are unaware of any studies which have measured Tenon's layer thickness by OCT.

## MATERIALS AND METHODS

This study was performed at Musgrove Park Hospital, Taunton, UK. A total of 67 eyes of 48 human subjects participated and were scanned using an optovue Mode RT100 version 2.0 OCT machine. Subjects were asked to look down and were scanned at the 12 o'clock position using cross-line CAM-L scans. The combined bulbar conjunctival and Tenon's

layer thickness was measured 3 mm above the superior limbus; a position which is commonly the site of the scleral fap dissection. Conjunctival and Tenon's layers appeared as a hyper-refective section as opposed to the hypo refective underlying scler a. Measurements were taken using the inbuilt review software.

**Fig. 1:**
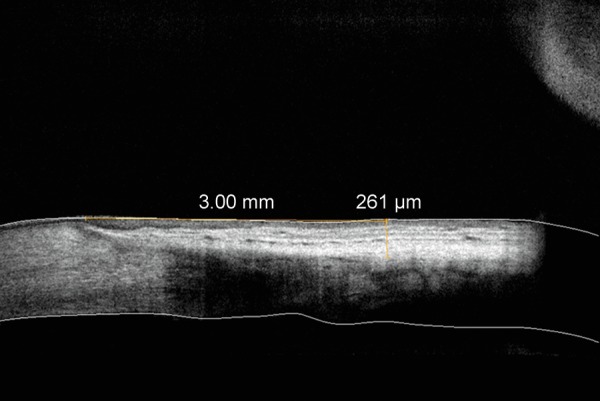
An OCT image of human bulbar conjunctiva and Tenon's capsule *in vivo*. The thickness measurement of conjunctiva and Tenon's 3 mm from the superior limbus is 261 mm

The limbus was identified on the scan and from this a 3 mm measurement was taken superiorly. From this point the thickness measurement was made (Fig. 1). Scans were taken by 3 examiners who initially studied several scans together to ensure good interobserver correlation.

We certify that all applicable institutional and governmental regulations concerning the ethical use of human volunteers were followed during this research.

**Graphs 1A and B:**
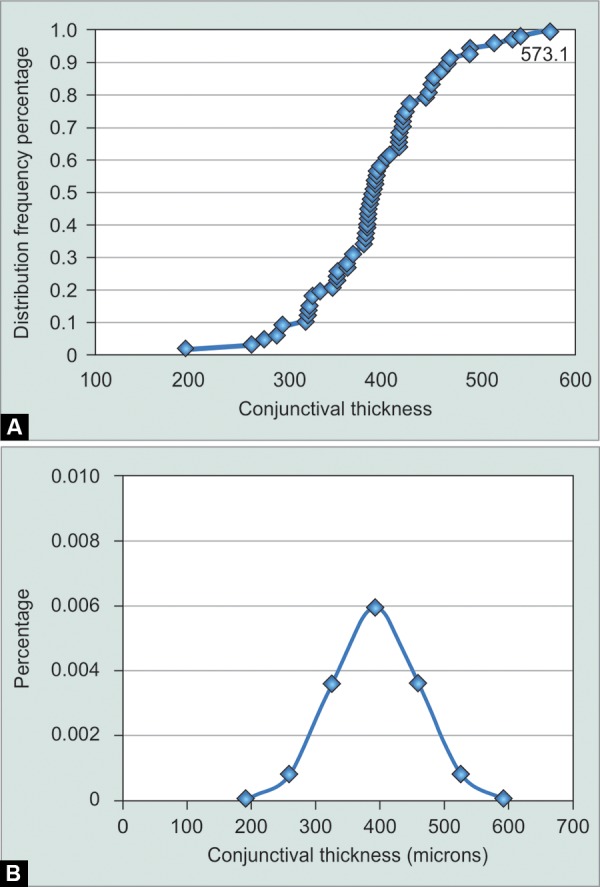
The distribution of combined conjunctival and Tenon's layer thickness in each subject: (A) Frequency distribution, (B) Normal distribution

## RESULTS

The age of the subjects ranged between 23 and 91 years. There were 20 males and 28 females. The mean combined conjunc tival and Tenon's layer thickness was 393 ± 67 μm (± standard deviation) ranging from 194 to 573 μm (Graphs 1A and B). There were 29 male eyes and 38 female eyes; the mean thickness of the male eyes was 398 ± 69 μm, and of the female eyes 390 ± 66 μm.

**Graph 2:**
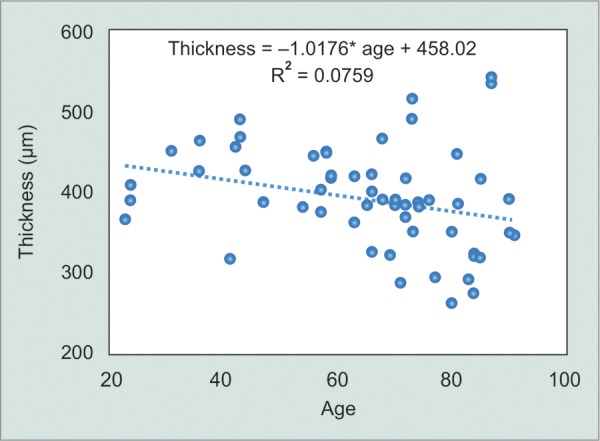
Linear regression analysis. R^2^ = 0.76 with a standard error of 63.75 (p = 0.032)

Eyes over age 40 showed a mean thickness of 389 μm and under age 40 a mean thickness of 417 μm (1 eye age unknown). However, there was a very small sample (10 eyes) under age 40. A Pearson correlation reveals a negative and low correlation between age and conjunctival thickness (–0.276, p = 0.032). Single linear regression analysis was performed which shows R^2^ = 0.76 with a standard error of 63.75 (p = 0.032) (Graph 2).

## DISCUSSION

This study appears to support our clinical observations that conjunctival and Tenon's layer thickness measurements vary significantly between individuals.

The average measurement we found of the conjunctiva and Tenon's layers combined was slightly less than that found by Zhang et al and Cho et al.^12,15^ There are several reasons why this could be the case (1) Tenon's could give a different measurement on OCT than when measured *in vitro*; Zhangetal^15^ found that their value for epithelial conjunctival thickness on OCT differed from that found by confocal microscopy. Furthermore, different OCT machines may gived iffering measu rements, (2) The conjunctiva could be a different thick ness superiorly, (3) Due to measurement error, e.g. in the accuracy locating the junction of Tenon's/sclera. However, we have shown the variability in the measu rement between individuals.

It is recognized that the final bleb morphology is highly depen dent on the surgical technique.^16^ However, even when the same technique is used by a single surgeon the final morphology may vary enormously, with some patients developing bleb encapsulation and/or failure whilst others develop thin walled avascular blebs which are at higher risk of leak and bleb related infection. Bleb failure occurs when there is a pronounced fibrotic reaction from Tenon's capsule fibroblasts.^17^ Perhaps avascular blebs are more likely when there are insufficient fibroblasts.

We postulate that these measurements could help predict the risk of postoperative bleb encapsulation and/or failure; with patients most at risk perhaps having thicker conjunctival and Tenon's layer measurements. Also, perhaps those with thinner measurements could be at risk of conjunctival break down and as a result aqueous leaks, avascular blebs and there fore bleb related infection.

Nuzzi et al^18^ studied patients on topical medical therapy for primary open angle glaucoma and found an increase in thickness and number of epithelial layers, increase in fibroblast density in subepithelial and deep connective tissue in conjunctival biopsies. Therefore, it may also be help ful to include information on topical glaucoma medication in further studies as this could affect the thickness measurements.

Clinically antifibrotics (MMC and 5-FU) reduce fibro-blast proliferation in the subconjunctival space and in Tenon's capsule to prevent episcleral fibrosis; however, they do increase the likelihood that blebs will be thin, cystic or avas cular; characteristics that increase the risk of conjunc-tival breakdown and aqueous leakage.^19^ Francis et al^19^ found that conjunctiva exposed to antifibrotics had fewer layers of epithelial cells than conjunctiva with no prior exposure.

Further, prospective studies could help establish limits for which patients should or should not receive intraoperative antimetabolites and /or Tenon's layer excision.
